# Evaluation of an alternative technique to optimize direct bonding of orthodontic brackets to temporary crowns

**DOI:** 10.1590/2176-9451.20.4.057-062.oar

**Published:** 2015

**Authors:** Francilena Maria Campos Santos Dias, Célia Regina Maio Pinzan-Vercelino, Rudys Rodolfo de Jesus Tavares, Júlio de Araújo Gurgel, Fausto Silva Bramante, Melissa Nogueira Proença Fialho

**Affiliations:** 1 MSc in Orthodontics, Universidade Ceuma, São Luís, Maranhão, Brazil; 2 Assistant professor, Universidade Ceuma, Department of Orthodontics, São Luís, Maranhão, Brazil; 3 PhD in Oral Rehabilitation, Universidade de São Paulo (USP), School of Dentistry, Bauru, São Paulo, Brazil; 4 PhD in Orthodontics, Universidade Ceuma, São Luís, Maranhão, Brazil; 5 Associate Professor, Master’s Program in Odontology, Universidade Ceuma, São Luís, Maranhão, Brazil.; 6 MSc in Orthodontics, Universidade Ceuma, São Luís, Maranhão, Brazil

**Keywords:** Orthodontic brackets, Dental bonding, Shear bond strength, Temporary dental restoration

## Abstract

**OBJECTIVE:**

To compare shear bond strength of different direct bonding techniques of orthodontic brackets to acrylic resin surfaces.

**METHODS:**

The sample comprised 64 discs of chemically activated acrylic resin (CAAR) randomly divided into four groups: discs in group 1 were bonded by means of light-cured composite resin (conventional adhesive); discs in group 2 had surfaces roughened with a diamond bur followed by conventional direct bonding by means of light-cured composite resin; discs in group 3 were bonded by means of CAAR (alternative adhesive); and discs in group 4 had surfaces roughened with a diamond bur followed by direct bonding by means of CAAR. Shear bond strength values were determined after 24 hours by means of a universal testing machine at a speed of 0.5 mm/min, and compared by analysis of variance followed by post-hoc Tukey test. Adhesive remnant index (ARI) was measured and compared among groups by means of Kruskal-Wallis and Dunn tests.

**RESULTS:**

Groups 3 and 4 had significantly greater shear bond strength values in comparison to groups 1 and 2. Groups 3 and 4 yielded similar results. Group 2 showed better results when compared to group 1. In ARI analyses, groups 1 and 2 predominantly exhibited a score equal to 0, whereas groups 3 and 4 predominantly exhibited a score equal to 3.

**CONCLUSIONS:**

Direct bonding of brackets to acrylic resin surfaces using CAAR yielded better results than light-cured composite resin. Surface preparation with diamond bur only increased shear bond strength in group 2.

## Abstract (pt)

**OBJETIVO:**

comparar a resistência ao cisalhamento de diferentes técnicas para colagem direta de braquetes ortodônticos em superfície de resina acrílica.

**MÉTODOS:**

sessenta e quatro (64) discos de resina acrílica ativada quimicamente (RAAQ) foram divididos aleatoriamente em qautro grupos: grupo 1 = colagem com resina composta fotopolimerizável (adesivo convencional); grupo 2 = superfície abrasionada com broca diamantada e colagem com resina composta fotopolimerizável; grupo 3 = colagem com RAAQ (adesivo alternativo); grupo 4 = superfície abrasionada com broca diamantada e colagem com RAAQ. A resistência ao cisalhamento foi avaliada 24h após a colagem, utilizando-se uma máquina universal de ensaios, operando a uma velocidade de 0,5mm/min, e comparada por meio da análise de variância, seguida pelo teste *post-hoc* de Tukey. O índice de adesivo remanescente (IAR) foi mensurado e comparado entre os grupos por meio dos testes Kruskal-Wallis e Dunn.

**RESULTADOS:**

os grupos 3 e 4 demonstraram resistência ao cisalhamento significativamente maior do que os grupos 1 e 2. Os grupos 3 e 4 apresentaram resultados similares. O grupo 2 apresentou melhores resultados do que o grupo 1. Na análise do IAR, observou-se predominância do escore 0 para os grupos 1 e 2 e do escore 3 para os grupos 3 e 4.

**CONCLUSÕES:**

a colagem do braquete em superfície de resina acrílica utilizando-se a RAAQ apresentou melhores resultados do que a colagem com resina composta fotopolimerizável. A abrasão da superfície com broca diamantada apenas aumentou a resistência ao cisalhamento no grupo 2.

## INTRODUCTION

In recent decades, there has been a growing concern for esthetics, in addition to increased life expectancy of individuals. As a result, the number of adult patients seeking orthodontic treatment has significantly increased.^1^ Patients seek orthodontists for personal reasons, including esthetic or functional improvements of occlusion, or because other dentists refer them to have dental movements carried in order to aid different restorative procedures.

In many cases, adult patients use definitive or temporary crowns.^2^ Temporary crowns are commonly used to protect tissues and render dental position stable before definitive crown manufacture.^3^Additionally, they also contribute to reestablish both esthetics and function during rehabilitation treatment.

Whenever a patient has temporary crowns, definitive restoration before orthodontic treatment is not recommended due to occlusal changes resulting from dental movement. In these cases, the orthodontist must bond or band the accessories on the surface of temporary material. Banding is recommended for posterior teeth; however, in the anterior region, direct bonding of accessories is used due to the poor esthetic aspect of bands.

During orthodontic movement of healthy teeth, bracket bonding on tooth enamel is part of orthodontic routine. However, bonding brackets on acrylic surfaces of temporary crowns is critical and presents high bonding failure rates. Frequent rebonding procedures hinders the advance of mechanotherapy, thereby contributing to increase treatment time, costs and chair-side time.^4^ Therefore, these procedures are undesirable for both orthodontist and patient.

Among the different types of material available to manufacture temporary crowns, chemically activated acrylic resin (CAAR) is the most commonly used^5 ^due to being inexpensive, easily manipulated and allowing repair, adjustment and relining, all of which may prove necessary during treatment. Additionally, this resin is resistant to oral function, including mastication; supports orthodontic forces and does not damage dental crown surface during debonding.

Despite the advantages of using CAAR as a temporary restorative material, its surface has lower bond strength values in comparison to what is clinically acceptable, when testing orthodontic accessories bonding.^5^ Various studies have compared different types of material used to manufacture temporary crowns,^6^^-^ 10 different methods of preparing the provisional material surface before direct bracket bonding^5^^,^ 11 ^,^ 12 and different adhesives.^7^ However, studies assessing the use of CAAR as a material for direct bonding of orthodontic brackets are scarce.

Composite resin is a common adhesive used for direct bracket bonding regardless of the surface (tooth or restoration). However, this type of material yields results lower than what is clinically acceptable when brackets are bonded to provisional acrylic resin material.^5^ Thus, it has been speculated that the use of an alternative adhesive could optimize the results. Therefore, the objective of this study was to test the following null hypothesis: there is no difference in shear bond strength values of different direct bonding techniques (varying adhesive material and surface treatment) of orthodontic brackets to acrylic resin surfaces.

## MATERIAL AND METHODS

Sample size calculation was carried out by means of the statistical program SAS, version 9.1.3. (SAS Institute Inc., Cary, USA). The following parameters were adopted: shear bond strength value of 5.3 ± 3.3 MPa;^6^ significance level set at 5%; power of test of 80%; and effect size equal to 1. A sample size (n) of 12 specimens in each group was then determined. As a precaution, n = 16 was adopted.

Sixty-four CAAR Duralay discs (Dental Mfg. Co, Worth, USA) were prepared using rigid polyvinyl chloride (PVC) cylinders as matrix. Resin was prepared following the manufacturer's instructions (volume ratio of 3 : 1). After manipulation, the material was poured into the PVC rings until 3-mm thickness was reached. The remaining space in the PVC ring was filled with colorless CAAR also prepared according to the manufacturer's instructions (volume ratio of 2.5 : 1). To homogenize the bonding surfaces, CAAR surfaces were finished and polished with silicon carbide sandpaper in decreasing order of roughness (400 and 600). Specimens were then randomly divided into four groups (Table 1).

**Table 1.** Description of groups.

Groups	Surface preparation and adhesive agent
1	Acid etching, application of primer and bracket bonding using light-cured composite resin.
2	Surface roughened with diamond bur, acid etching, application of primer and bracket bonding using light-cured composite resin.
3	Acid etching, application of monomer and bracket bonding using CAAR.
4	Surface roughened with diamond bur, acid etching, application of monomer and bracket bonding using CAAR.

In group 1 (conventional adhesive without surface treatment = control group), surface was polished for 10 seconds with extra-fine pumice and rubber prophylactic cups with the aid of a low-speed hand piece. It was then rinsed with water and dried with oil-free air spray for 30 seconds. Subsequently, 37% gel phosphoric acid was applied for 30 seconds, followed by copious washing for 30 seconds and drying with oil-free air spray for 15 seconds. A thin coat of Transbond XT primer (3M Unitek, Monrovia, Calif., USA ) was applied, followed by drying with a brief air spray and light-curing for 20 seconds. Stainless steel 14.79-mm^2^brackets (Standard Edgewise maxillary central incisors, Morelli Ortodontia, Sorocaba, SP, Brazil) were bonded to the surface prepared with Transbond XT light-cured composite resin (3M Unitek , Monrovia, Calif). Brackets remained in the manufacturer's package until immediately before bonding and were handled with bonding tweezers to avoid contamination of the bonding base.

Brackets were positioned at the center of specimens and then firmly pressed during 5 seconds, so as to obtain a fine layer of bonding material.^13^Excess material was carefully removed by means of an exploratory probe. Light curing was performed with a light intensity of 450 mW/cm^2^ measured by a radiometer. The duration of light incidence was 20 seconds, 10 seconds on each side (mesial and distal), following the manufacturer's instructions.

**Figure 1.**
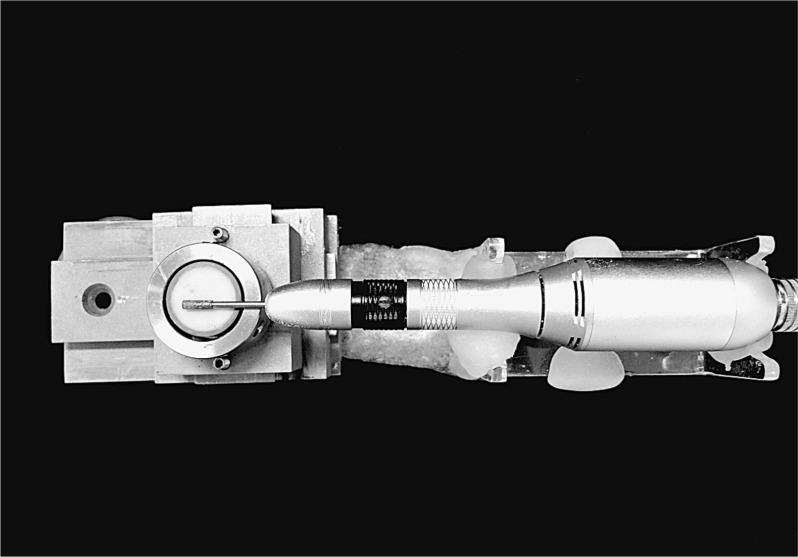
Surface preparation with a diamond bur

In group 2 (conventional adhesive with surface treatment), surface was initially roughened with a cylindrical diamond bur, medium granulation (PM 82. Vortex; São Paulo, Brazil). The bur was positioned parallel to the surface of the specimen with a rotation speed of 4,000 rpm (Fig 1). Brushing movements were made with the bur over the specimens, using a device for standardization. Subsequently, prophylaxis, acid etching and direct bracket bonding were performed as described for group 1.

In group 3 (alternative adhesive without surface treatment), after prophylaxis, 37% gel phosphoric acid was applied for 30 seconds, followed by copious rinsing for 30 seconds and drying with oil-free air spray for 15 seconds. Afterwards, CAAR monomer was applied with the aid of a brush, and direct bracket bonding was performed with Duralay resin applied to the base of brackets with a brush, according to the powder/liquid technique; subsequently, brackets were positioned. After excess material removal, self-curing of CAAR was carried out.

Group 4 (alternative adhesive with surface treatment) was subjected to the same roughening procedure described for group 2. The direct bonding procedure was carried out as described for group 3.

**Figure 2.**
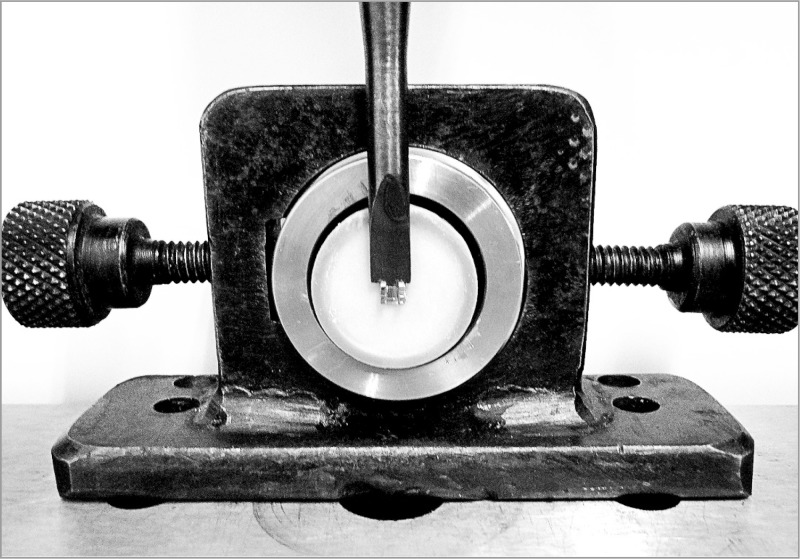
Specimen ready to be tested

After the bonding procedure, specimens were immediately stored in distilled water, in a bacteriological incubator at 37 ± 1 ^o^C. Tests were performed after 24 hours.^14^ Shear bond strength was assessed by means of a universal testing machine, with a 50-kg load cell, operating at a speed of 0.5 mm/min^15^ (Fig 2).

A single previously calibrated operator performed all bonding procedures. Laboratory tests were conducted by another operator who was blind with respect to the technique used for bonding.

After bracket debonding, the surfaces were analyzed by two examiners using a magnifying glass under 5 x magnification (Illuminated Magnifier, Fujian, China), so as to determine the adhesive remnant index (ARI). Scores recommended by Artur and Bergland were used.^16^ They range from 0 to 3, as follows: 0 = no adhesive left on dental enamel; 1 = less than half adhesive left on dental enamel; 2 = more than half adhesive left on dental enamel; and 3 = all adhesive left on dental enamel, including impression of the bracket mesh. Although they were developed to assess the enamel surface, in the present study, these scores were applied to assess acrylic resin surface.^17^^,^ 18

Shapiro-Wilk test confirmed that data followed normal distribution. Analysis of variance (ANOVA) and *post-hoc* Tukey test were used to compare groups. Data regarding ARI were analyzed by means of Kruskal-Wallis and Dunn tests for multiple comparisons. Significance level was set at *p* < 0.05. Statistical analyses were performed with SAS software version 9.1.3 (SAS Institute Inc., Cary, USA).

## RESULTS

Results revealed that groups 3 and 4, in which brackets were bonded by means of CAAR, had significantly greater shear bond strength values than groups 1 and 2, in which brackets were bonded with light-cured composite resin. Groups 3 and 4 yielded similar results (Table 2).

**Table 2.** Means, standard deviation (SD), minimum and maximum values in MPa and comparison between groups.

Groups	Mean ± SD*	Min. value	Max. value
Group 1	1.38 ± 0.40^a^	0.82	2.14
Group 2	4.37 ± 1.14^b^	2.55	6.61
Group 3	12.19 ± 1.58^c^	9.7	15.2
Group 4	12.41 ± 1.96^c^	10.19	16.22

* Different letter suggest statistically significant difference (Tukey test).

The surface prepared with diamond bur yielded better bonding only for the group bonded with light-cured composite resin (Table 2).

Regarding ARI, groups 1 and 2 predominantly exhibited a score equal to 0, whereas groups 3 and 4 predominantly exhibited a score equal to 3. Multiple comparisons demonstrated that groups 1 and 2 and groups 3 and 4 were similar. However, ARI differed significantly when comparing groups 1 and 2 with groups 3 and 4 (Table 3).

**Table 3.** ARI scores and comparison between groups.

Groups	Scores				Median*
	0	1	2	3	
Group 1	16	0	0	0	0^a^
Group 2	13	1	0	2	0^a^
Group 3	0	0	2	14	3^b^
Group 4	0	0	0	16	3^b^

* Different letter suggest statistically significant difference.

## DISCUSSION

Direct bracket bonding to temporary material must be of good quality to support orthodontic forces applied during dental movement, as well as masticatory forces. The present study was developed to test an alternative technique using CAAR as adhesive to improve shear bond strength in these cases. Results suggest that the null hypothesis was rejected because significant differences were observed among the techniques analyzed.

The methods used in this study were based on the literature about bonding of artificial acrylic resin teeth to the base of complete dentures^9^ and surfaces of temporary material bonded by means of light-cured composite resin^6^^,^ 7 ^,^ 11 ^,^ 12 due to shortage of studies on this subject.

Before the direct bonding procedure was carried out, specimens had their surfaces treated with 37% phosphoric acid. Phosphoric acid is a bactericidal agent that increases the energy of dental enamel surface by removing non-reactive hydroxyapatite crystals and the acquired pellicle, thereby transforming the surface into a highly porous tissue.^19^ Although the acid does not alter the original topography of acrylic resin, this substance was used to promote cleaning of debris generated during the preparation of the bonding surfaces.^7^^,^ 12 A previous study conducted by Thean, Chew and Goh^20^ reported that removal of contaminants, such as saliva and material residues, was more important than the mechanical preparation of surfaces. Bonding failure can occur if the surface is contaminated before the bonding procedures.

In the present study, groups 3 and 4 yielded the best shear bond strength values. The results obtained for the alternative technique using CAAR as adhesive showed values that could be considered as clinically acceptable.^21^ Studies demonstrating satisfactory bonding forces between orthodontic brackets and temporary crowns are scarce in the literature. Various studies assessing direct bonding of brackets onto temporary material have reported bond strength values below what is clinically acceptable.^11^^,^ 12

Shear bond strength values for groups 3 and 4 were greater than those obtained by Chay *et al,* 5 Maryanchik *et al,* 6 Blakey and Mah^11^and Masioli *et al.* 12 The main difference between our study and the aforementioned ones was the adhesive used to bond brackets to provisional crowns. However, when compared to group 1, the values reported by certain authors^5^^,^ 11 were greater. This result is most likely related to the use of different procedures in the preparation of the tested surfaces.

In groups 3 and 4, it is believed that moistening the test surface with monomer provided an additional measure to improve effectiveness of the acrylic resin/acrylic resin chemical interaction.^10^

Despite the high values obtained for groups 3 and 4, it is important to emphasize that *in vivo* and *in vitro* studies comparing bond strength demonstrate that the values obtained* in vivo* are significantly lower than those obtained *in vitro.* 22

Orthodontists commonly use diamond burs to prepare bonding surfaces on provisional crowns.^5^ As in a previous study,^7^ surface preparation with burs was performed following a systematic protocol conducted by a single operator. Minor differences in the surfaces, if present, most likely did not affect the results obtained. Masioli *et al* 12 assessed roughness of surfaces prepared with burs and demonstrated reasonable uniformity with a variation coefficient below 30%.

When the groups were compared in terms of surface treatment, results revealed that shear bond strength values differed significantly between groups 1 and 2, with group 2 exhibiting higher values than group 1. It is speculated that this result occurred because, after curing, the composite resin requires macromechanical retentions to become attached to other material. Composite resin does not chemically react with the acrylic resin of temporary crowns, which gives support to the better bonding values yielded by roughened surfaces compared with surfaces without preparation.^12^ It is further speculated that the tertiary amines present in CAAR composition can inhibit adequate polymerization of light-cured composite resins, thereby impairing bonding between different types of material. Inhibition could also explain the lower bond strength values observed in groups 1 and 2 compared with the values observed in groups 3 and 4. It is emphasized that the strength values obtained for groups 1 and 2 were lower than what is considered as clinically acceptable.^21^

Groups 3 and 4, bonded with CAAR, exhibited similar strength values. It is speculated that this result occurred because the test surface had a short aging time, and the mechanical properties of the material were not altered after bonding.^5^

The predominant ARI score for groups 1 and 2 was 0. This result indicates that when light-cured composite resin was used as adhesive, there was no effective bond between the bracket and the tested surface. In groups 3 and 4, in which CAAR was used as bonding material, the predominant ARI score was 3, which can be explained by the fact that CAAR underwent chemical interaction with a new material of the same composition after being moistened with the monomer.^10^ These findings suggest that groups 3 and 4 exhibited adequate bonding between the specimen and CAAR used as bonding material.

Results suggest that when there is a need to bond accessories to acrylic resin surfaces, the orthodontist can effectively use CAAR as adhesive and monomer before the procedure.

## CONCLUSIONS

Direct bonding of orthodontic brackets to acrylic resin surfaces using CAAR was effective in increasing shear bond strength.

Surface preparation by means of a diamond bur increased shear bond strength only in the group bonded with light-cured composite resin; however, the values obtained for this group were lower than what is clinically acceptable.

## References

[1] Pabari S, Moles DR, Cunningham SJ (2011). Assessment of motivation and psychological characteristics of adult
orthodontic patients. Am J Orthod Dentofacial Orthop.

[2] Wassell RW, Steele JG, Welsh G (1998). Considerations when planning occlusal rehabilitation: a review of the
literature. Int Dent J.

[3] Yannikakis SA, Zissis AJ, Polyzois GL, Caroni C (1998). Color stability of provisional resin restorative
materials. J Prosthet Dent.

[4] Pasquale A, Weinstein M, Borislow AJ, Braitman LE (2007). In-vivo prospective comparison of bond failure rates of 2 self-etching
primer/adhesive systems. Am J Orthod Dentofacial Orthop.

[5] Chay SH, Wong SL, Mohamed N, Chia A, Yap AUJ (2007). Effects of surface treatment and aging on the bond strength of
orthodontic brackets to provisional materials. Am J Orthod Dentofacial Orthop.

[6] Maryanchik I, Brendlinger EJ, Fallis DW, Vandewalle KS (2010). Shear bond strength of orthodontic brackets bonded to various
aesthetic pontic materials. Am J Orthod Dentofacial Orthop.

[7] Rambhia S, Heshmati R, Dhuru V, Lacopino A (2009). Shear bond strength of orthodontic brackets bonded to provisional
crown materials utilizing two different adhesives. Angle Orthod.

[8] Cardash HS, Applebaum B, Baharav H, Liberman R (1990). Effect of retention grooves on tooth-denture base bond. J Prosthet Dent.

[9] Chung RWC, Stanford JW, Serio A (2011). Properties of self-curing denture base resins. J Adv Prosthodont.

[10] Cunningham JL, Benington IC (1999). An investigation of variables that may affect the bond between plastic
teeth and denture base resin. J Dent.

[11] Blakey R, Mah J (2010). Effects of surface conditioning on the shear bond strength of
orthodontic brackets bonded to temporary polycarbonate crowns. Am J Orthod Dentofacial Orthop.

[12] Masioli DLC, Almeida MAO, Masioli MA, Almeida JRM (2011). Assessment of the effect of different surface treatments on the bond
strength of brackets bonded to acrylic resin. Dental Press J Orthod.

[13] Machado CT, Borges BCD, Araujo GJR, Santos AJS, Dametto FR, Pinheiro FHSL (2012). Influence of adhesion promoters and curing-light sources on the shear
bond strength of orthodontic brackets. Indian J Dent Res.

[14] Endo T, Ozoe R, Shinkai K, Aoyagi M, Kurokawa H, Katoh Y (2009). Shear bond strength of brackets rebonded with a fluoride-releasing and
recharging adhesive system. Angle Orthod.

[15] Isber H, Ambrosio AR, Carvalho PEG, Valle-Corotti KM, Siqueira DF (2011). Comparative in vitro study of the shear bond strength of brackets
bonded with restorative and orthodontic resins. Braz Oral Res.

[16] Artun J, Bergland S (1984). Clinical trials with crystal growth conditioning as an alternative to
acid-etch enamel pretreatment. Am J Orthod.

[17] Northup RG, Berzins DW, Bradley TG, Schuckit W (2007). Shear bond strength comparison between two orthodontic adhesives and
self-ligating and conventional brackets. Angle Orthod.

[18] Bishara SE, Soliman MM, Oonsombat C, Laffoon JF, Ajlouni R (2004). The effect of variation in mesh-base design on the shear bond strength
of orthodontic brackets. Angle Orthod.

[19] Vieira TI, Valença AMG, Santiago BM, Gondim BLC (2011). Antimicrobial activity of phosphoric acid associated or not associated
with 2% chlorhexidine over dental biofilm bacteria. Int J Dent.

[20] Thean HP, Chew CL, Goh KI (1996). Shear bond strength of denture teeth to base: a comparative
study. Quintessence Int.

[21] Reynolds PR (1975). A review of direct orthodontic bonding. Br J Orthod.

[22] Hajrassie MKA, Khier SE (2007). In-vivo and in-vitro comparison of bond strengths of orthodontic
brackets bonded to enamel and debonded at various times. Am J Orthod Dentofacial Orthop.

